# Adherence to Mediterranean Diet Measured through Medi-Lite Score and Obesity: A Retrospective Study

**DOI:** 10.3390/nu13062007

**Published:** 2021-06-10

**Authors:** Monica Dinu, Giuditta Pagliai, Sofia Lotti, Ilaria Giangrandi, Barbara Colombini, Francesco Sofi

**Affiliations:** 1 Department of Experimental and Clinical Medicine, University of Florence, 50121 Florence, Italy; giuditta.pagliai@gmail.com (G.P.); sofia.lotti@unifi.it (S.L.); barbara.colombini@unifi.it (B.C.); francesco.sofi@unifi.it (F.S.); 2 Unit of Clinical Nutrition, Careggi University Hospital, 50139 Florence, Italy; giangrandii@aou-careggi.toscana.it

**Keywords:** Mediterranean diet, adherence, Medi-Lite, obesity

## Abstract

We recently developed and validated a questionnaire to measure adherence to the Mediterranean diet, called Medi-Lite. The aim of this study was to assess the accuracy of the Medi-Lite adherence score in relation to obesity status. A total of 208 patients who attended the Clinical Nutrition Unit of Careggi University Hospital, Florence, were included in this retrospective analysis. Of them, 126 (45%) had abdominal obesity (110 F; 16 M). The mean adherence score, calculated through the Medi-Lite questionnaire, was 9.5 ± 2.2, with significantly (*p* < 0.001) lower values in patients with abdominal obesity (8.9 ± 1.9) than those without abdominal obesity (10 ± 2.2). Logistic regression analysis adjusted for age and sex showed that the Medi-Lite score determined significant protection (−28%) against the risk of abdominal obesity for every one-unit increase in the total score (OR 0.72, 95% CI 0.63–0.82; *p* < 0.001). Looking for cut-off values that denote increased risk of having abdominal obesity, we observed that patients who scored ≤9 had a significantly increased risk (OR 3.21, 95% CI 1.91–5.39; *p* < 0.001). Adherence to the Mediterranean diet assessed through the Medi-Lite score was found to be associated with abdominal obesity. In particular, patients who reported a score of ≤9 had a 3.5-fold times higher risk of having abdominal obesity than those who scored >9.

## 1. Introduction

The Mediterranean diet is globally recognised as a healthy dietary pattern for the maintenance of health status and for the prevention of major chronic degenerative diseases [1]. In order to identify a method for estimating adherence to this dietary model, research in recent decades has focused on summarising the diet through a single index or score resulting from the combination of different food components [2]. In 2014, our group developed an adherence score based on data from the scientific literature, called Medi-Lite, which allows the assessment of adherence to the Mediterranean diet through simple questions about the consumption of the main food components of the Mediterranean diet [3]. The Medi-Lite questionnaire was validated in 2017 [4], and several studies using the questionnaire in different populations have recently been published [5,6,7,8].

Despite the recognition of the validity of Medi-Lite in the scientific literature, some problems remain in the identification of cut-off values associated with the risk profile of the patient who undergoes the questionnaire. In particular, there is a lack of information on the possible association between adherence to the Mediterranean diet analysed through the Medi-Lite score and the presence of a pathological state such as obesity.

In this context, the aim of the present study was to evaluate whether the Mediterranean diet adherence score obtained through the Medi-Lite questionnaire can be associated with the presence of obesity and to possibly identify the optimal cut-off for defining obesity risk.

## 2. Materials and Methods

A retrospective evaluation of patients who were admitted for their first clinical evaluation at the Clinical Nutrition Unit of the University Hospital of Careggi, Florence, Italy, from January to April 2021, was conducted. The study population included a total of 280 patients (208 females and 72 males) with a mean age of 51.3 years old (median: 53; range: 16–83 years).

Data were obtained through structured standard questionnaires administered by trained personnel. Information on socio-demographic variables, lifestyle (including physical activity and tobacco smoking), and comorbidities were collected. Weight and height were measured using a stadiometer. Body mass index (BMI) was calculated as the weight (kg)/height (m^2^). Patients were classified as overweight if their BMI was between 25 and 30 kg/m^2^, and obese if their BMI was ≥30 kg/m^2^ [9]. Class I obesity was defined as BMI between 30 and 35 kg/m^2^, class II obesity as between 35 and 40 kg/m^2,^ and class III obesity as BMI ≥40 kg/m^2^. Waist circumference was measured in the horizontal plane at the midpoint between the iliac crest and the lowest rib. High waist circumference values were identified as values >102 cm in males and >88 cm in females [10], and a high percentage of fat mass was defined as values above 25% in men and 35% in women, as suggested by the World Health Organization (WHO) [11]. Body composition was determined by a bioelectrical impedance analysis device (TANITA, model TBF-410).

The study was approved by the Ethics Committee (CEAVC 18353/OSS) of the Tuscany Region, Careggi University Hospital, Florence, and adhered to the principles of the Declaration of Helsinki and the Data Protection Act. Informed consent was obtained from all subjects involved in the study.

### 2.1. The Medi-Lite Adherence Score

The Medi-Lite adherence score consists of nine items that assess the daily consumption of fruit, vegetables, cereals, meat and meat products, dairy products, alcohol, and olive oil, and the weekly consumption of legumes and fish [3]. For each food group composing the score, there are three categories of consumption. These categories have been defined based on data available in the literature in relation to adherence to the Mediterranean diet and health status. For foods typical of the Mediterranean diet (fruit and vegetables, cereals, legumes, and fish), 2 points are assigned to the highest consumption category, 1 to the middle category, and 0 to the lowest category. As to olive oil, 2 points are assigned for regular use, 1 for frequent use, and 0 for occasional use. Foods not typical of the Mediterranean diet (meat and meat products, dairy products) are scored as follows: 2 points to the lowest category, 1 to the middle category, and 0 to the highest category of consumption. Finally, 2 points are assigned to the middle consumption category of alcohol (1–2 alcohol units/day), 1 to the lowest category (1 alcohol unit/day), and 0 to the highest category (>2 alcohol units/day). The final score ranges from 0 (low adherence) to 18 (high adherence to the Mediterranean diet).

### 2.2. Statistical Analysis

Statistical analysis was performed using the statistical package PASW 27.0 for Macintosh (SPSS Inc., Chicago, IL, USA). Results were expressed as mean ± standard deviation (SD) or median and min–max range, as appropriate. Differences between groups were estimated using the unpaired Student’s *t*-test. The chi-square test was used to test for proportions. Correlation analyses were performed through Spearman’s correlation test. Logistic regression analysis was conducted to identify the risk of abdominal obesity, evaluated as a composite index identified by the concomitant presence of BMI ≥30 kg/m^2^, waist circumference > 102 cm in males or >88 cm in females, and fat mass percentage > 25% in males or >35% in females, according to the different scores of the Medi-Lite adherence score adjusted for age and gender. Results were reported as odds ratio (OR) and 95% confidence intervals (CI). A *p*-value < 0.05 was considered statistically significant.

## 3. Results

### 3.1. Characteristics of the Study Population

Anthropometric measurements and body composition of the study population are reported in Table 1.

The mean BMI of the patients included in the study was 31.5 ± 4.3 kg/m2 (range: 25.2–48.2 kg/m^2^), with significantly higher values in females than males (*p* < 0.05). Taking BMI as the sole indicator of obesity, more than half of the study population was classified as obese (n = 162, 57.9%), while the rest were classified as overweight (n = 118, 42.1%). No significant differences were observed for sex within the different groups of obesity classes. Regarding the other body composition parameters, 234 (83.6%) and 249 patients (88.9%), respectively, reported waist circumference and fat mass percentage values above the reference values. For waist circumference, there was a significantly higher prevalence in females than in males (*p* < 0.0001).

To assess the presence of abdominal obesity, we used a composite index that included both the presence of BMI > 30 kg/m^2^ and the concomitant presence of waist circumference and fat mass percentage above the reference values. Using this composite index, 126 patients (45%) were found to have abdominal obesity (110 F; 16 M).

### 3.2. Medi-Lite Score

The Medi-Lite score in the study population was 9.5 ± 2.2 (median: 10, range 5–15) with slightly but not significantly higher values in males (9.6 ± 2.1) than in females (9.3 ± 2.3). Correlation analyses showed significant inverse correlations between Medi-Lite score and anthropometric measurements such as body weight (R = −0.38; *p* < 0.0001), BMI (R = −0.41; *p* < 0.0001), fat mass in kilograms (R = −0.29; *p* < 0.0001), fat mass in percentage (R = −0.15; *p* = 0.03) and waist circumference (R = −0.28; *p* < 0.0001).

**Figure 1:**
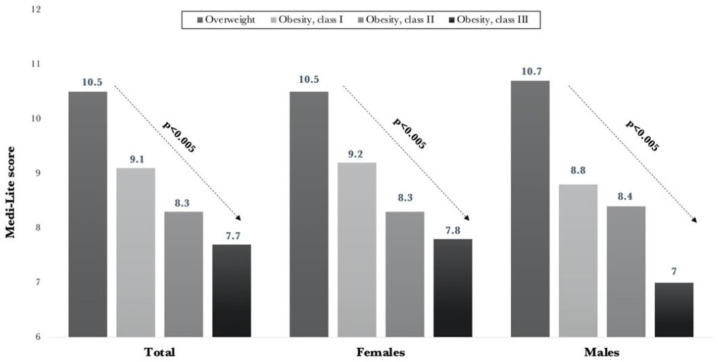
Medi-Lite values according to BMI categories.

Grouping patients according to BMI categories, we observed a significantly (*p* < 0.0001) lower adherence score in patients with higher BMI values in both males and females (Figure 1).

Similarly, grouping patients by the presence of abdominal obesity assessed through the composite index, patients with abdominal obesity reported significantly (*p* < 0.0001) lower values (8.9 ± 1.9) than patients without abdominal obesity (10 ± 2.2).

### 3.3. Medi-Lite Score and Risk of Abdominal Obesity

Logistic regression analysis adjusted for age and sex showed that the Medi-Lite score determined significant protection (−28%) against the risk of abdominal obesity for every one-unit increase in the total score (OR 0.72, 95% CI 0.63–0.82; *p* < 0.001). Subsequently, to identify the cut-off values of the Medi-Lite adherence score in determining the risk of abdominal obesity, we conducted the analysis with the composite obesity index as the dependent variable and the Medi-Lite score as the independent variable.

**Figure 2:**
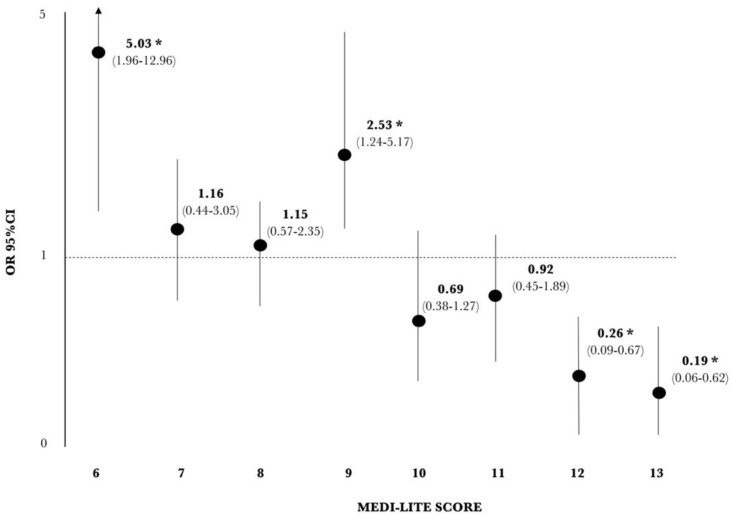
Logistic regression analysis for the risk of abdominal obesity according to the Medi-Lite score; * *p* < 0.05 adjusted for age and gender.

A significantly increased risk of having abdominal obesity was reported for patients who scored 6 and 9 points (Figure 2), with a significantly increased risk for those who scored ≤9 points (OR 3.21, 95% CI 1.91–5.39; *p* < 0.001).

On the other hand, patients reporting ≥10 points showed a trend towards a reduced risk of having abdominal obesity, with statistical significance reached for patients who scored 12 and 13 points. Indeed, patients reporting a score of ≥12 showed a significantly reduced risk of having abdominal obesity (OR 0.19, 95% CI 0.09–0.41; *p* < 0.0001).

## 4. Discussion

In this retrospective analysis of an unselected group of patients who underwent a clinical evaluation at our Clinical Nutrition Unit, we demonstrated that the Medi-Lite adherence score was significantly associated with the presence of clinical parameters suggestive of obesity status. A one-unit increase in the score was associated with a 28%-reduced risk of having a composite index of abdominal obesity, measured as the concomitant presence of high values of BMI, waist circumference, and percentage of fat mass. In addition, patients who reported a score of 9 or less had a 3.2-fold increased risk of being identified as clinically obese, compared with those who reported a higher score, while those who scored ≥12 had a reduced risk. These cut-off values may be useful for clinicians to identify patients at higher risk of developing the disease, and for individuals to self-assess the quality of their diet.

The Mediterranean diet is universally recognised as the ideal dietary pattern to maintain health status and reduce the risk of chronic diseases in the general population [12,13]. Despite this universal recognition, many countries in the Mediterranean area are experiencing a process of ‘westernisation’ of food habits, and a progressive loss of adherence to this dietary model [14,15]. With the aim of measuring adherence to the Mediterranean diet, especially in relation to the risk of developing diseases, many Mediterranean diet adherence scores have been created [2]. Some of these scores focused mainly on their usefulness as a tool that can be applied to epidemiological studies, while others focused on their clinical utility in identifying subjects or patients at higher risk of developing chronic degenerative diseases [16].

In 2014, with the aim of creating a tool useful for clinicians in an outpatient setting, we developed the Medi-Lite adherence score [3]. It is based on the concept that the ideal portions of the typical food groups of the Mediterranean diet can be defined based on all prospective cohort studies that have investigated the association between adherence to the Mediterranean diet and overall mortality. Obtaining these values from studies published in the literature has the advantage, compared to other adherence tools, of assessing the association between the Mediterranean diet and mortality by following data from specific populations rather than recommendations of guidelines or opinions of experts in the field. Another advantage is its ease of use since the score consists of nine simple questions that can be carried out in a short time even by non-experts or directly by the patient himself. As previously reported, the use of short questionnaires to assess adherence to the Mediterranean diet is useful to examine the effect of this dietary pattern on health outcomes and to provide immediate feedback on diet quality [17,18].

Despite these advantages, Medi-Lite has a limitation in that no cut-off values have been identified so far. To date, the data derived from its administration have been used through the analysis of tertiles or quartiles of its distribution in the specific population of interest [5,6,7,8,19,20]. In the present study, we identified possible threshold values that are associated with an increased risk of this obesity, a growing concern in both industrialised populations and developing countries [21]. To identify the clinical presence of obesity, we used a composite index that included BMI values ≥30, as well as waist circumference, the clinical parameter that best discriminates the presence of abdominal fat mass [22], and the percentage of fat mass. By applying this composite index, we found that greater adherence to the Mediterranean diet was significantly associated with a lower prevalence of abdominal obesity. This is in line with previous studies that have investigated the possible relationship between Mediterranean diet adherence indexes and metabolic disorders such as obesity, diabetes, and hypertension [18,22,23,24]. The analysis of 7447 participants in the PREDIMED trial showed a strong inverse association between the 14-item Mediterranean Diet Adherence Screener (MEDAS) and abdominal obesity [18]. Similarly, higher scores on the Italian Mediterranean Index were associated with reduced weight gain and reduced increase in waist circumference in volunteers recruited in EPIC-Italy [24]. Finally, two recent cross-sectional studies conducted on Japanese [25] and Gulf [26] populations have shown that a two-point increase in the MedDiet score decreases the risk of becoming overweight or obese.

With regard to the two cut-off points that can be used to give an estimate of the risk of obesity after completion of the Medi-Lite questionnaire, we found that subjects reporting a score of ≤9 had a 3.2-fold increased risk of obesity than those reporting higher scores. On the contrary, those reporting scores >12 had a significantly reduced risk of obesity. The inverse association between Medi-lite score and abdominal obesity is not surprising as several mechanisms can explain the protective effect of the Mediterranean diet against weight gain. Firstly, the Mediterranean Diet contains high amounts of fibre-rich foods that induce satiety, decrease the absorption of macronutrients, and enhance the production of short-chain fatty acids by intestinal bacteria [27]. Secondly, the nutritional adequacy that can be achieved by following this dietary pattern may contribute to improved glycaemic control, higher insulin sensitivity, reduced postprandial inflammation, lower oxidative stress [28], and reductions in other mechanisms associated with abdominal fat deposition [29].

Some limitations can be identified in the present study. Firstly, the lack of common criteria for defining typical and non-typical foods of the Mediterranean diet meant that the available adherence scores included components considered most suitable, depending on the objectives of the study and the available data. In the case of Medi-Lite, the food groups comprising the score were defined based on previous prospective cohort studies investigating the association between adherence to the Mediterranean diet and overall mortality. We are aware that this approach may be debatable, as are all other approaches, but to date, there is no gold-standard method for defining adherence to the Mediterranean diet. Secondly, the sample is not very large. Studies on a larger number of patients are needed before the results can be extended and the associations found validated. A further limitation is that the analysis is retrospective and therefore not free from possible methodological bias. Prospective longitudinal studies are needed to understand the true predictive role of the Medi-Lite adherence score in modulating the risk of obesity and related diseases. Finally, the patients examined were selected from those referred to a specialised nutrition centre and are therefore not representative of the general population. We cannot exclude the possibility that these patients were more likely to have a poor dietary profile associated with the degree of obesity. Despite all these limitations, the present study has the strength of being the first study to evaluate the association between the Medi-Lite adherence score and the clinical condition of obesity and to identify cut-off values that can be used in the ambulatory setting at the patient level.

## 5. Conclusions

In conclusion, the results of this retrospective analysis of a group of patients referred to our Clinical Nutrition Unit showed a significant association between the Medi-Lite adherence score and the clinical condition of obesity, highlighting threshold values that identify an increased risk of obesity. These results, if confirmed in larger studies, may be clinically useful for those using this score as a tool to assess the nutritional quality and adherence to the Mediterranean diet.

## Figures and Tables

**Table 1:** Anthropometric characteristics and body composition.

	Males (n = 72)	Females (n = 208)
Body weight	96.1 ± 16.6	84.2 ± 13.8
BMI, kg/m^2^	31.3 ± 4.5	32.2 ± 3.9
Overweight, n (%)	24 (33.3)	94 (45.2)
Obesity class I, n (%)	32 (44.4)	78 (37.5)
Obesity class II, n (%)	15 (20.8)	28 (13.5)
Obesity class III, n (%)	15 (20.8)	28 (13.5)
Obesity class IV, n (%)	1 (1.4)	8 (3.8)
WC, cm	105.7 ± 8.1	99.2 ± 9.1
WC > 102 cm M; >88 cm F	42 (58.3)	192 (92.3)
Fat mass, kg	32 ± 9.6	34.9 ± 9.6
Fat mass, %	32.8 ± 6.6	40.4 ± 4.9
Fat mass > 25% M; >35% F	63 (87.5)	186 (89.4)

Data are reported as mean ± SD or as n (%); BMI = body mass index; F = females; M = males; WC = waist circumference.

## Data Availability

Data will be made available on request.
